# Progression from mild cognitive impairment to dementia in Alzheimer's disease: Whole cortex voxelwise functional connectivity analysis with multivariate distance matrix regression

**DOI:** 10.1177/13872877261477016

**Published:** 2026-08-12

**Authors:** Luiz Kobuti Ferreira, Eric Westman, Lars-Olof Wahlund, Rosaleena Mohanty, for the Alzheimer's Disease Neuroimaging Initiative

**Affiliations:** 1 Division of Clinical Geriatrics, Center for Alzheimer Research, Department of Neurobiology, Care Sciences and Society, 27106 Karolinska Institutet, Stockholm, Sweden; 2 Centre for Psychiatry Research, Department of Clinical Neuroscience, 27106 Karolinska Institutet, & Stockholm Health Care Services, Region Stockholm, Sweden; 3 The Ageing Epidemiology Research Unit, School of Public Health, 4615 Imperial College London, London, UK

**Keywords:** Alzheimer's disease, functional connectivity, functional neuroimaging, mild cognitive impairment

## Abstract

**Background:**

Differentiating individuals with mild cognitive impairment who convert to dementia due to Alzheimer's disease (MCI-C) from those who do not convert (MCI-NC) is increasingly important. Functional connectivity (FC) derived from resting state functional MRI (rs-fMRI) has been investigated as a potential biomarker. However, improved data analysis strategies are needed. One underexplored approach is pairwise voxel-to-voxel analysis.

**Objective:**

To describe differences in FC between amyloid positive MCI-C and MCI-NC using a whole-cortex voxel-to-voxel pairwise approach.

**Methods:**

Baseline rs-fMRI from the Alzheimer's Disease Neuroimaging Initiative was retrieved for amyloid positive MCI participants. Voxel-to-voxel, pairwise, cortical FC was computed. Multivariate distance matrix regression was used to identify voxels presenting FC patterns that were significantly different between MCI-C and MCI-NC.

**Results:**

21 MCI-C and 28 MCI-NC were included. The primary analysis with voxel-level threshold at p < 0.001 combined with cluster-level p < 0.05 yielded no significant results. At voxel-level p < 0.01 and the same cluster-level threshold, three significant clusters on the right visual cortex were found. These clusters, however, were not robust to head motion, fMRI protocol and additionally clinical or biological severity.

**Conclusions:**

Progression from Alzheimer-related MCI to dementia was not significantly associated with FC in the primary analysis. However, a less stringent threshold yielded FC differences in the occipital lobe in alignment with previous studies but were not robust to methodological and biological covariates between groups. Our findings highlight the need for larger samples and careful control of covariates to identify robust FC alterations related to dementia conversion.

## Introduction

The neuropathological changes in Alzheimer's disease (AD) begin many years before symptom onset and strategies aiming at modifying the course of the disease are thought to be more effective if implemented before the development of clinical dementia.^1–4^ Identifying those that in the future will convert to dementia is important when selecting samples for trials of disease-modifying agents.

Individuals with mild cognitive impairment (MCI) are at increased risk of progressing to dementia. Among those with MCI with AD pathology such as amyloid positivity, the risk is even higher.^5^ However, even among these individuals many do not convert to dementia during the follow up.^6^ Incorporating connectivity-based biomarkers of neurodegeneration beyond cognitive and amyloid markers could be a potential strategy to select people with MCI who have the most potential to benefit from disease-modifying agents.

A potential candidate to further refine the risk-stratification of individuals are functional brain changes since those are supposed to precede even asymptomatic neurostructural changes.^2^ Functional connectivity (FC) refers to the statistical relationships or temporal correlations between the activity of different brain regions.^7^ It is a growing subfield of neuroimaging in neurodegenerative disorders, and it has been found to be altered in asymptomatic stage with amyloid deposition, MCI and dementia due to AD.^8,9^ With the relatively widespread access to functional magnetic resonance imaging (fMRI) and the feasibility of resting-state acquisitions, FC could represent a potential tool if we overcome the challenge of analyzing FC data in order to generate clinically useful results. As pointed by others, fMRI-based connectivity measures require substantial improvement to become useful biomarkers in clinical trials, and one possible avenue is the advancement of data analysis methods.^10^

A number of studies have found variable or non-significant FC changes when studying individuals with MCI who later convert to dementia.^11–16^ These previous studies encompass different methodological choices representing a number of limitations such as limited focus on specific brain networks or seeds,^13,17–21^ the need to binarize the connectivity matrix,^15,16,22,23^ and externally derived brain parcellation schemes that reduce spatial resolution while also enforcing an arbitrary, rigid number of parcels.^12,16,21,23–26^ Traditionally, voxelwise approaches have been used in fMRI studies and opened fruitful research avenues.^27–29^ However, this approach has been underexplored in the field of fMRI-based connectivity, often due to the higher and often unfeasible computational demands of voxel-to-voxel pairwise connectivity analysis.^30^ Voxelwise approaches often lead to high-dimensional features which can introduce challenges of overfitting and statistical instability especially in smaller sample sizes.

In this study, we compared voxelwise FC patterns between MCI individuals with AD pathology who remained cognitively stable compared to those who converted to dementia using a multivariate approach that preserves spatial resolution and avoids reliance on predefined parcellations or networks. By adopting a voxel-to-voxel pairwise connectivity approach using multivariate distance matrix regression (MDMR) we performed an exploratory analysis involving the whole cortex, without restrictions regarding specific cortical regions or networks and no need to enforce external parcellation schemes or loss of spatial resolution. We focused on the brain cortex since this is where the AD-related pathological processes have been traditionally described and where most FC changes have been studied and reported.^8,31^ MDMR allowed the association between conversion to AD and the full set of pairwise relationships between a given voxel and all other cortical voxel to be computed in a single statistical step.

## Methods

### Alzheimer's Disease Neuroimaging Initiative

Data used in the preparation of this article were obtained from the Alzheimer's Disease Neuroimaging Initiative (ADNI) database (adni.loni.usc.edu). The ADNI was launched in 2003 as a public-private partnership, led by Principal Investigator Michael W. Weiner, MD. The original goal of ADNI was to test whether serial MRI, positron emission tomography (PET), other biological markers, and clinical and neuropsychological assessment can be combined to measure the progression of MCI and early AD. The current goals include validating biomarkers for clinical trials, improving the generalizability of ADNI data by increasing diversity in the participant cohort, and to provide data concerning the diagnosis and progression of AD to the scientific community. For up-to-date information, see adni.loni.usc.edu.

### Ethical approval

The ADNI study protocol was approved by institutional review boards of all participating institutions, and written informed consent was obtained from all participants or their legal representatives at study sites.

### Participants, criteria for inclusion

Inclusion criteria for all participants were: 1) diagnosis of MCI at baseline assessment; 2) the baseline resting state (rs)-fMRI was performed no more than 90 days from the clinical baseline assessment, 3) the baseline rs-fMRI passed ADNI's quality control, had a repetition time (TR) close to 3 s (between 2900 ms and 3100 ms, consistent with prior studies^21,32^ in order to include participants with comparable rs-fMRI protocols) with a minimum of 140 fMRI timepoints (volumes), and 4) a positive amyloid status on amyloid-PET with either ^18^F-florbetaben or ^18^F-florbetapir performed within one year from the baseline rs-fMRI to ensure that all participants harbored this AD-related pathology. Amyloid imaging positivity was calculated and determined by ADNI's amyloid imaging methods as described in the document “UC Berkeley Amyloid PET Methods”, available at https://ida.loni.usc.edu/: the standardized uptake value ratio (SUVR), using the whole cerebellum as the reference region was thresholded for amyloid positivity at 1.08 for ^18^F-florbetaben and 1.11 for ^18^F-florbetapir. When comparing groups or including amyloid as a covariate, the quantification of amyloid deposition was based on ADNI's centiloid scale, retrieved from the ADNI website for each participant. Briefly, the centiloid scale aims at transforming the degree of amyloid deposition to a 0-to-100 scale, anchored by young controls and typical patients with AD. ADNI's method to compute the centiloid scale is described in the document “Amyloid PET Processing Methods” available at https://adni.loni.usc.edu/ and in-depth information can be found at Klunk et al. (2015).^33^

All included participants had a MCI diagnosis at baseline according to ADNI's criteria: memory concern as reported by the participant, partner or clinician, abnormal memory function assessed with the Logical Memory II subscale, Delayed Paragraph Recall, Paragraph A from the Wechsler Memory Scale–Revised, adjusted for education (≤ 11 for 16 or more years of education; ≤ 9 for 8–15 years of education; ≤ 6 for 0–7 years of education), Mini-Mental State Exam (MMSE) score between 24 and 30 (inclusive), Clinical Dementia Rating = 0.5 with Memory Box score at least 0.5 and general cognition and functional performance sufficiently preserved such that the person did not fulfill criteria for diagnosis of dementia due to AD.

The ADNI's databases with longitudinal diagnostic information were downloaded on 17 January 2025.

*Selection of MCI converters (MCI-C)*. Participants with MCI at baseline who progressed to dementia due to AD within three years. Participants who converted to dementia after three years were not included. On one hand this time limit potentially excludes participants with slower cognitive decline that are nevertheless on the path towards dementia, thus representing a clinically relevant group. On the other hand, this time window is consistent with previous studies^11,13,34^ and reflects a balance between sample size and preserving the clinical utility of baseline measures. Those who, after converting to dementia, reverted back to MCI or to normal controls were not included. Further, participants who were at any time considered to have dementia due to other etiologies were excluded.

*Selection of MCI non-converters (MCI-NC)*. All ADNI participants were filtered to identify those that had MCI at baseline and never converted to dementia during follow-up. Only those who were followed for at least three years were included.

Since the MCI stage is clinically and biologically very heterogeneous, we limited the study sample to amyloid positive cases in both groups to increase biological specificity for AD-related conversion.

### MRI acquisition

MRI was acquired using Philips, GE and Siemens 3 T scanners.

*Protocols for rs-fMRI*. ADNI 2 Philips 3T: TE 30 ms, TR 3000 ms, flip angle 80, scan duration 10 min, isotropic voxel size 3.125 mm, 48 slices. ADNI 3 Philips 3T: TE 30 ms, TR 3000 ms, flip angle 90, scan duration 10 min, isotropic voxel size 3.39 mm, 48 slices. ADNI 3 GE 3T: TE 30 ms, TR 3000 ms, flip angle 90, scan duration 10 min, isotropic voxel size 3.4 mm, 48 slices. ADNI 3 Siemens 3T: TE 30 ms, TR 3000 ms, flip angle 90, scan duration 10 min, isotropic voxel size 3.4 mm, 48 slices. Participants were asked to keep their eyes open and fixated on a point during rs-fMRI acquisition.

*Protocols for T1 (structural MRI)*. ADNI 2 Philips 3T: TR 6.8 ms, TE 3.1 ms, flip angle 9, voxel size 1 × 1 mm, slice thickness 1.2 mm, 170 slices. ADNI 3 Philips 3T: TR 6.5 ms, TE 2.9 ms, flip angle 9, voxel size 1 × 1 × 1 mm, 211 slices. ADNI 3 GE 3T: TR 2.3 ms, TE 7.3 ms, flip angle 11, voxel size 1 × 1 mm, slice thickness 1.2 mm, 256 slices. ADNI 3 Siemens 3T: TR 2.3 ms, TE 2.98 ms, flip angle 9, voxel size 1 × 1 × 1 mm, 256 slices.

### Preprocessing

The baseline structural T1 MRI and rs-fMRI from each participant was retrieved from the ADNI's database and transformed from DICOM to NIfTI format using dcm2niix.^35^ The T1 images underwent skull-stripping and spatial normalization to the MNI space using sswarper2 from AFNI.^36^ Individually tailored masks from the lateral ventricles and the white matter were obtained from each raw T1 images using Freesurfer's recon-all^37^ followed by AFNI's @SUMA_Make_Spec_FS (https://afni.nimh.nih.gov/Suma).

AFNI's afni_proc.py was used to preprocess rs-fMRI, as following: 1) removal of the first two TRs, 2) time series despiking, 3) slice time correction, 4) one-step spatial transformation encompassing both coregistration and normalization to MNI (applying the output of sswarper2), 5) spatial smoothing with a gaussian kernel (4 mm FWHM), 5) one-step noise removal related to motion (six motion parameters and their first derivatives), signal from lateral ventricles (the three principal components of the time series from the ventricles extracted using the lateral ventricle mask) and local white matter (voxel-specific signal from the white matter by applying the white matter mask and AFNI's ANATICOR^38^) and 6) censoring of frames immediately preceding and following instances of excessive motion, calculated by the Euclidean norm of the first derivative of the six motion parameters > 0.5. For each participant, head motion during rs-fMRI acquisition was quantified as the mean Euclidean norm of the first temporal derivatives of the six motion parameters.

Parallel processing with GNU Parallel^39^ was used in a number of pre- and postprocessing scripts.

Due to excessive motion, three participants were excluded (one for motion exceeding 5 mm between any two frames and two due to more than 30% of frames being censored due to motion).

All preprocessing parameters were chosen a priori, without any adjustment after reaching the results, as previously recommended.^40^

### Cortical mask

Since the brain cortex is where AD-related pathology has been traditionally described and where most FC changes have been studied and reported,^8,31^ we used a cortical mask to select the voxels undergoing analysis. A group-wise cortical mask was built by retrieving each participant's grey matter parcellation from AFNI's @SUMA_Make_Spec_FS and filtering for only cortical regions. Each mask was spatially normalized to MNI space and blurred with a gaussian filter at 2 mm FWHM. The average of all participants’ masks was binarized using a threshold of 0.5 and resampled to the rs-fMRI grid (voxel size 3 × 3 × 3 mm). Only voxels containing valid times series from all participants were included in the common cortical mask, resulting in a sample specific cortical mask with 22,859 voxels.

### Voxelwise cortical functional connectivity

**Figure 1.**
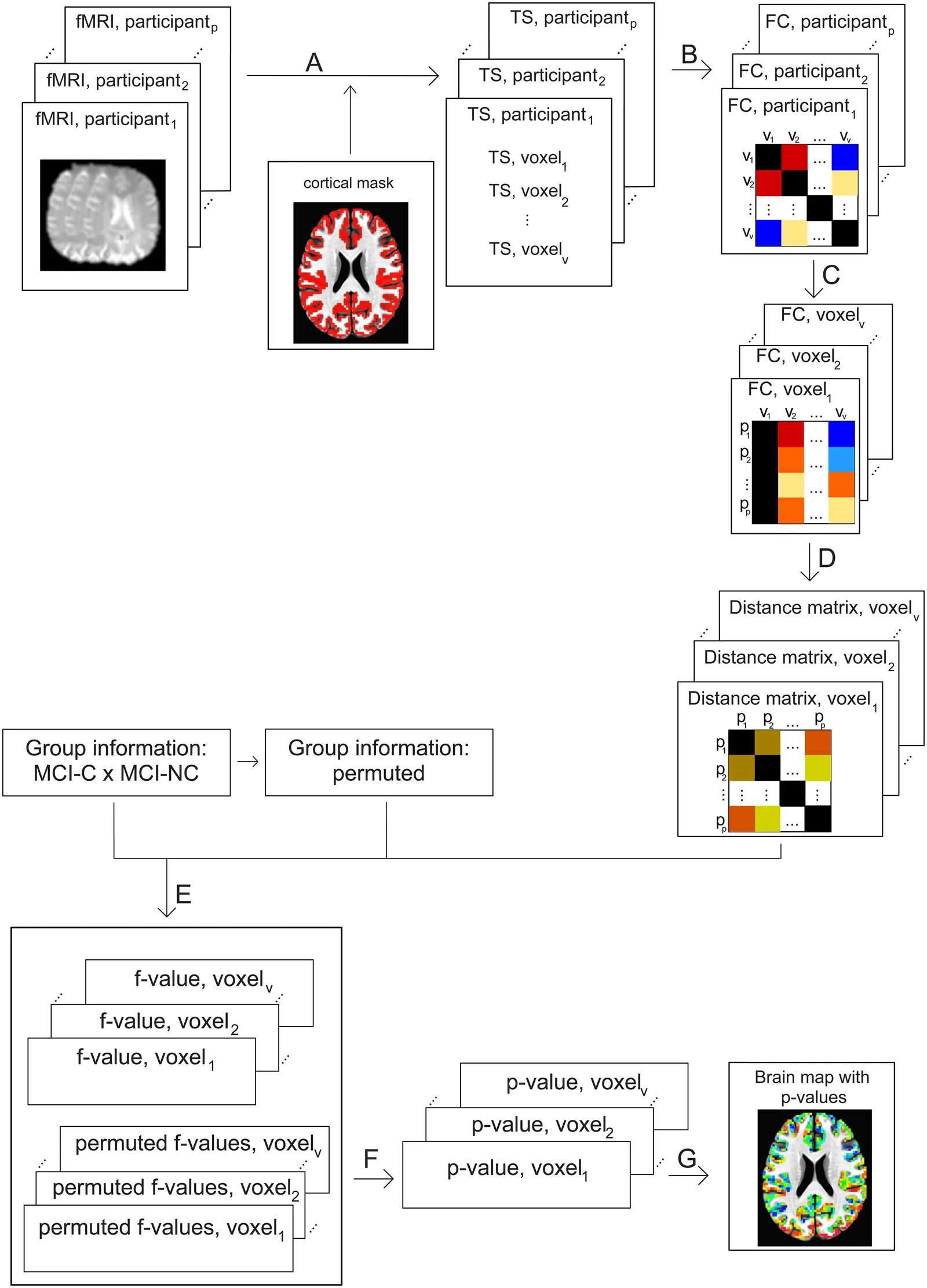
Overview of the analysis (A) rs-fMRI undergoes preprocessing and then the time series of each cortical voxel is extracted using the common mask. (B) The correlations between the time series are calculated, resulting in *p* square matrices of size *v* x *v* where v is the number of cortical voxels and *p* is the number of participants. (C) For each voxel, gather its connectivity pattern with all other voxels, stacking this pattern for all participants. This results in one *p* x *v* matrix for each voxel. (D) For each voxel, calculate the L1-distances between connectivity patterns among all possible pairs of participants, creating one distance matrix of size *p* x *p* for each voxel. (E) By randomly permuting the information about group membership (MCI-C x MCI-NC), calculate permuted f-values. (F) Estimate the p-value for each voxel. (G) Map back the p-values to their cortical voxels. FC: functional connectivity; fMRI: functional magnetic resonance imaging; TS: time series.

For each participant, the time series from each voxel within the cortical mask was extracted, thus retrieving 22,859 time series per participant (Figure 1A). The Pearson correlation coefficient between all possible pairs of voxels within the common cortical mask was calculated resulting in 261,255,511 correlation coefficients per participant (Figure 1B). These FC values underwent Fisher's r-to-z transformation before undergoing analysis via MDMR.

### Multivariate distance matrix regression

We aimed at comparing the voxelwise FC patterns between MCI individuals with AD pathology that remained cognitively stable compared to those who converted to dementia. The applied strategy: 1) does not require parcellating the brain or using regions of interest, thus preserving spatial resolution; 2) is multivariate in nature but without requiring dimensionality reduction steps such as the need to select (and discard) a number of components; 3) does not require the selection of specific cortical networks and is independent from assumptions about the spatial distribution or anatomical characteristics of such cortical networks and 4) is computationally feasible even when faced with high number of edges (connections) derived from pairwise voxel-to-voxel connectivity analysis.

In a voxelwise connectivity analysis, the connectivity pattern of each cortical voxel is composed by the pairwise measures of connectivity between this voxel and each other cortical voxel. This meant 22 859 values for each voxel. Thus, the high-dimensionality, low-sample-size problem arises when testing for the association between such a large set of connections and conversion to AD. Standard general linear models are limited in such cases due to an increased risk of overfitting and the extremely high number of multiple comparisons. One strategy is to apply multivariate strategies such as MDMR. With MDMR the association between phenotype and the high dimensional connectivity pattern from each voxel can be computed in a single statistical test.^30^ Moreover, MDMR uses all information in the distance matrix, with no need for dimensionality reduction or selection of components as in many other multivariate approaches.^41^

To evaluate whether patterns of FC were associated with future conversion, we applied the voxelwise MDMR approach for brain connectivity^30^ using the MDMR package for R (https://cran.r-project.org/web/packages/MDMR/index.html).^41^ The connectivity pattern of each cortical voxel (i.e., the connectivity values between this voxel and all other voxels within the cortical mask) was assembled for all participants into a *p* x *v* matrix where *p* is the number of participants (*p* = 49) and *v* is the number of voxels (*v* = 22,859). Since this is computed for each voxel, there are *v* matrices, each one of size *p* x *v* (Figure 1C). Due to computational memory constraints, the computations were performed one *p* x *v* matrix at a time. For each *p* x *v* matrix, a distance matrix of size *p* x *p* was computed using the Manhattan distance (L1) between each possible pair of participants (Figure 1D). We choose initially the Manhattan distance since it is considered more robust against outliers than Euclidean distance.^41^ MDMR then tested the association between group status (MCI-C versus MCI-NC) and each distance matrix using permutation testing. The F statistic resulting from the original relationship between group status and connectivity was compared to a null distribution of F-values derived from 10,000 permutations in which participant group assignments were randomized and a new F-value was computed (Figure 1E). This permutation step yields a p-value for each voxel, quantifying the likelihood of observing the association under the null hypothesis of no relationship between conversion to AD and the voxel's FC pattern (Figure 1F). After repeating this procedure for each voxel, the product is a voxelwise cortical significance map reflecting the association between conversion to AD and the pattern of connectivity at each voxel (Figure 1G). More information about MDMR in the context of voxelwise connectivity analysis can be found at Shehzad et al. (2014).^30^

### Statistical thresholding

The voxelwise p-values from MDMR were mapped back to their corresponding cortical voxels, yielding a three-dimensional cortical statistical map. AFNI's 3dClustSim and 3dClusterize were used to compute and apply cluster-size thresholds given a voxelwise p-value in order to control for multiple comparisons. As recommended by AFNI, the flag -acf was used in 3dClustSim (-acf parameters were 0.7233, 3.3899 and 12.1138) in order to compute non-Gaussian spatial autocorrelation function, which reduces false-positive rates when generating cluster-size thresholds compared to previous gaussian-based strategies.^42^ A voxel was considered part of a cluster only if at least one of its faces is in direct contact with the rest of the cluster (i.e., first-nearest neighbor clustering). A standard voxelwise threshold at p < 0.001 combined with a cluster-level p < 0.05, corrected for multiple comparisons was used primarily. We also used a post hoc less stringent threshold with voxel-level p < 0.01, while keeping a cluster-level p < 0.05, corrected for multiple comparisons. These are commonly applied thresholds in analysis with AFNI.^42^ Given the voxelwise and the cluster-level thresholds, 3dClustSim calculated the minimum cluster size threshold which was then applied to the images using 3dClusterize.

## Results

### Characteristics of the participants included in the study

**Figure 2.**
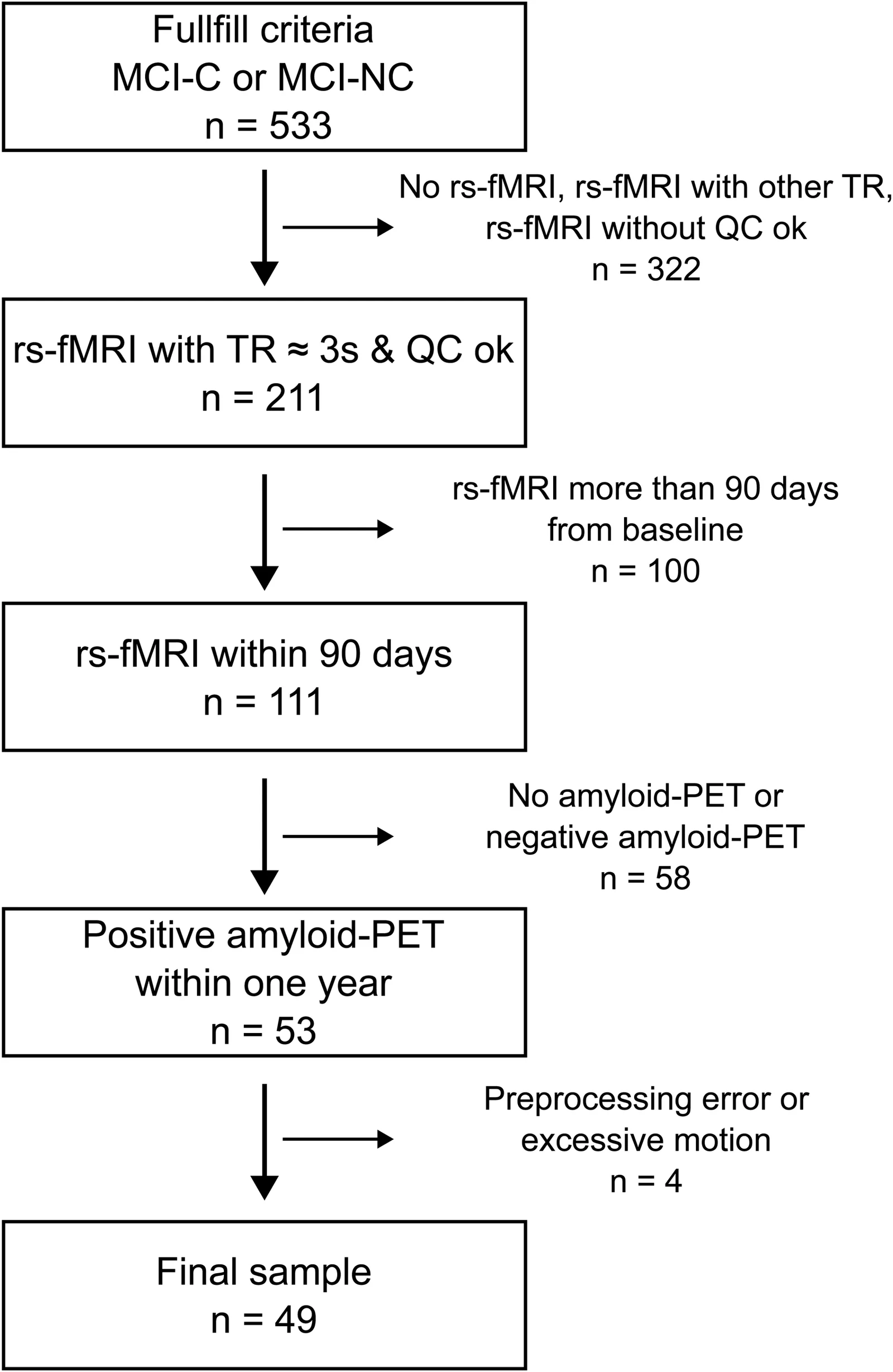
Flowchart, sample selection. MCI-C: participants with MCI at baseline who progressed to dementia due to AD within three years; MCI-NC: MCI at baseline and never converted to dementia during follow-up (minimal follow-up of three years); rs-fMRI: resting-state functional MRI; PET: positron emission tomography; QC: quality control as assessed by the Alzheimer's Disease Neuroimaging Initiative; TR: repetition time.

A flowchart showing the sample selection process is shown in Figure 2. An initial group of 21 MCI-C and 32 MCI-NC fulfilled the inclusion and exclusion criteria outlined in the Methods section. Due to errors in the conversion of one rs-fMRI from Dicom format (one participant) and excessive movement (three participants–see below, section Preprocessing), the final sample consisted of 21 MCI-C and 28 MCI-NC. The characteristics of the sample are shown on Table 1. The MCI-C had worse baseline cognitive performance (as measured with the MMSE) and higher amyloid burden on amyloid PET (p-values 0.03 and 0.02, respectively).

**Table 1.** Sample characteristics.

	MCI converters (n = 21)	MCI non-converters (n = 28)	p
Baseline age in years: mean (std dev)	74.1 (6.1)	73.1 (6.1)	0.58
Gender: Female	10 (48%)	12 (43%)	0.78
Education, years: mean (std dev)	16.9 (2.2)	16.3 (2.4)	0.36
Marital status: Partnered	19 (90%)	21 (75%)	0.27
*APOE4* carrier	13 (62%)	19 (68%)	0.76
Baseline MMSE: mean (std dev)	26.9 (1.6)	27.9 (1.5)	**0**.**03**
Motion in mm: mean (std dev)	0.15 (0.07)	0.13 (0.07)	0.49
Amyloid PET centiloid: mean (std dev)	87.9 (33.2)	65.3 (30.3)	**0**.**02**
Number of time frames (fMRI volumes) post-censoring, mean (std dev)	155.6 (33.8)	169.5 (31.6)	0.15
Time to conversion in days: mean (std dev)	597 (249)	–	–

Marital status: “partnered” were those married or in a ‘domestic partnership’ whereas unpartnered included ‘never married’, ‘widowed’ and ‘divorced’. Motion: average motion in millimeters during rs-fMRI per TR, before frame censoring (quantified as the mean Euclidean norm of the first temporal derivatives of the six motion parameters). The p-values were calculated with Welch's unequal variances t-tests for continuous variables and Fisher exact tests for the categorical variables. *APOE*: Apolipoprotein E gene; MCI: mild cognitive impairment; MMSE: Mini-Mental State Examination; PET: positron emission tomography.

### MDMR results

**Figure 3.**
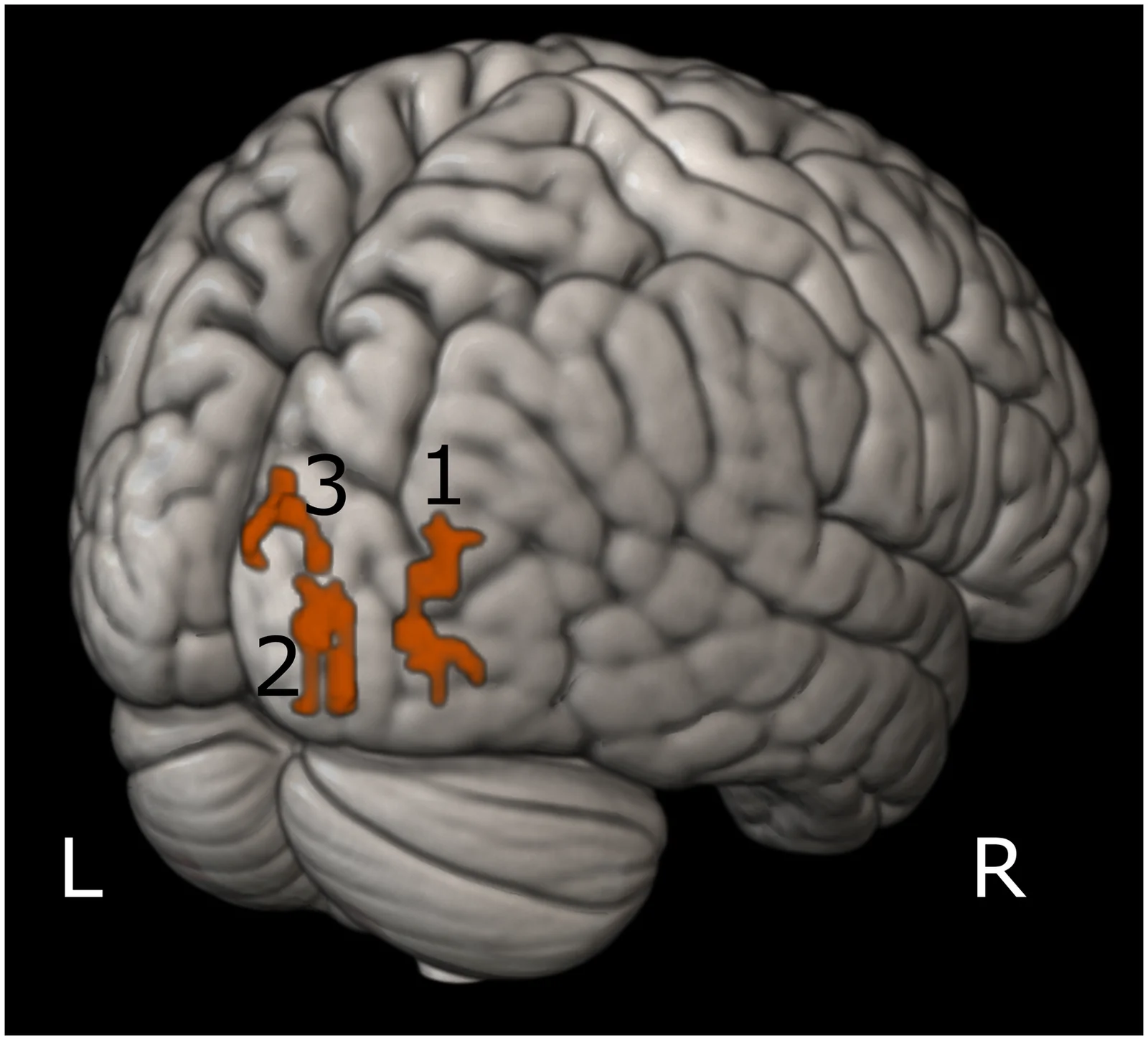
Regions with FC patterns associated with future conversion from MCI to dementia due to AD. Legend: Clusters with significantly different patterns of cortical FC at voxel-level p < 0.01 and cluster-level p < 0.05, corrected for multiple comparisons, from the MDMR analysis comparing MCI-C and MCI-NC (post hoc analysis with less strict voxel-level threshold). The clusters are identified with matching numbers as in Table 2 and are overlaid on a 3D-rendered MNI152 template using MRIcroGL (nitrc.org/projects/mricrogl). FC: functional connectivity; MCI-C: mild cognitive impairment–converters to Alzheimer's disease dementia; MCI-NC: mild cognitive impairment–nonconverters to Alzheimer's disease dementia; MDMR: multivariate distance matrix regression; L: left; R: right.

No significant clusters presenting FC patterns associated with future conversion from MCI to dementia due to AD were found when applying the primary, stringent statistical threshold (voxel-level p < 0.001 and cluster-level p < 0.05, controlled for multiple comparisons). In a post hoc analysis, keeping the cluster-level threshold at p < 0.05 (controlled for multiple comparisons) but with a less stringent voxel-level at p < 0.01 three clusters were identified on the right visual cortex, at V1 and V2 (Figure 3, Table 2). These voxelwise thresholds (p < 0.001 and 0.01) are in accordance with previous AFNI analysis.^42^

**Table 2.** MDMR result: clusters with differences in FC pattern between MCI-C and MCI-NC.

Cluster nr	Size (n voxels)	Brain area	Brodmann area	Peak coordinates (MNI)	p (FWE)
1	35	Postero-lateral visual cortex V2, R	18	40, −94, −8	0.0025
2	31	Postero-lateral visual cortex V1, R	17	26, −103, −8	0.0056
3	23	Postero-lateral visual cortex V2, R	18	23, −101, 13	0.0287

Significant clusters at voxel-level p < 0.01 and cluster-level p < 0.05. Peak coordinates in the LPI order, MNI space. MCI-C: mild cognitive impairment–converters; MCI-NC: non-converters; MDMR: multivariate distance matrix regression; R: right; p (FWE): p-value corrected for family-wise error at the cluster-level.

A post hoc analysis using Euclidean distance instead of Manhattan was also performed in order to verify if the results are similar using another distance metric. The result of this analysis is very similar to the one found using Manhattan distance and is presented in the Supplemental Material (Supplemental Table 1 and Supplemental Figure 1).

### Controlling for covariates

Since there were between-group differences in baseline performance on the MMSE and amyloid burden (amyloid PET, centiloid) and since head motion during fMRI and the fMRI protocol may have an impact on FC measurements, these covariates were included in post hoc MDMR analyses. Head motion was quantified using the mean Euclidean norm of the first temporal derivatives of the six motion parameters, as defined in the Methods section.

**Figure 4.**
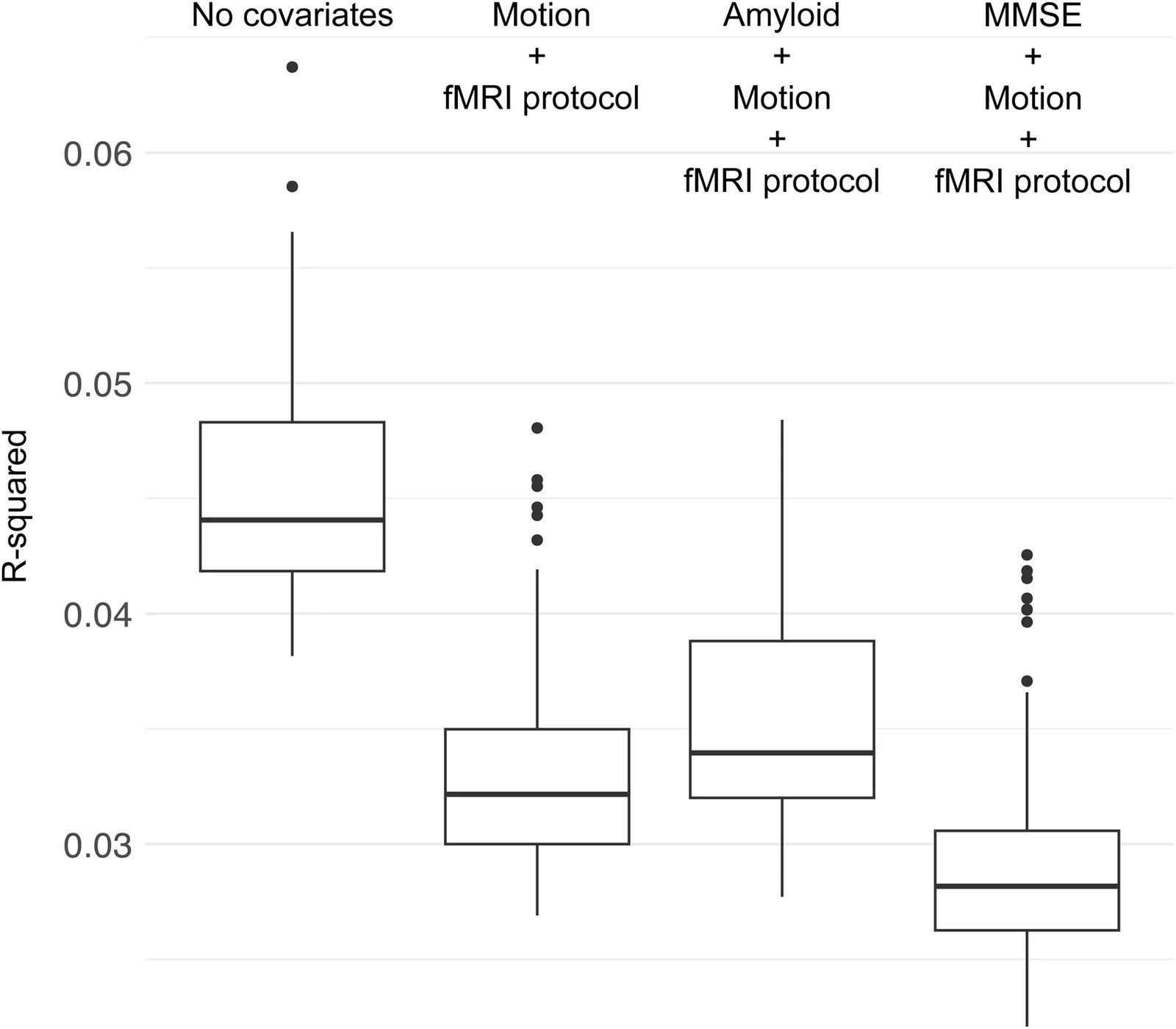
Voxelwise R-squared values from the MDMR analysis controlling for different covariates. Voxelwise MDMR analysis yields an R-squared value reflecting the effect size of the relationship between group (MCI-C, MCI-NC) and the voxel's pattern of FC. The boxplots show the distribution of R-squared values extracted from the 89 voxels from the three significant clusters (described on Table 2, Figure 3). The first boxplot from the left represents r-squared values from the MDMR analysis without covariates; the second resulted from the analyses controlling for head motion and fMRI protocol. In the third, amyloid centiloid was added while in the fourth (last to the right), MMSE was added. FC: functional connectivity; MCI-C: mild cognitive impairment, converters; MCI-NC: non-converters.

When controlling for head motion and fMRI protocol when performing MDMR, no statistically significant clusters were found both at the original voxel-level threshold (p < 0.001) and at the less strict threshold at p < 0.01 (both at cluster-level p < 0.05, controlled for multiple comparisons). We also included MMSE and degree of amyloid burden in two additional analyses (while also controlling for head motion and fMRI protocol) and found no significant clusters at both voxel-level thresholds (Table 3). The greatest reduction in effect size was observed when the association between FC and conversion was adjusted for MMSE, head motion and fMRI protocol, as estimated by averaging the r-squared value from the 89 voxels belonging to the three occipital clusters: a 35% decrease in the average R-squared, from 0.045 to 0.029. Just controlling for motion and protocol led to a decrease of 26%. Controlling for amyloid, motion and protocol led to the least decrease in mean r-squared from 0.045 to 0.036 (−20%). The distributions of the r-squared values when different covariates are controlled for are shown in Figure 4.

**Table 3.** MDMR results when controlling for covariates.

	Voxel-level threshold		
Covariates	p < 0.001	p < 0.01	Average R-squared
No covariates	No	Three clusters in R visual cortex (Table 2, Figure 3)	0.045
Head motion and fMRI protocol	No	No	0.033
Amyloid, head motion and fMRI protocol	No	No	0.036
MMSE, head motion and fMRI protocol	No	No	0.029

All analyses were thresholded at a cluster-level p < 0.05, corrected for multiple comparisons (family-wise correction). Average R-squared: from each voxel belonging to the significant clusters from Table 2, the R-square reflecting the variance from the distance FC matrix that is predicted by group membership was extracted and the average across voxels computed (89 voxels from the clusters described in Table 2 and shown in Figure 3). MDMR: multivariate distance matrix regression; MMSE: Mini-Mental State Examination; R: right.

## Discussion

This study investigated the cortical voxelwise differences in FC between nonconverters and converters from MCI to dementia due to AD. Our a priori statistical threshold did not yield significant results, while a less stringent threshold, still corrected for multiple comparisons, yielded findings in the right visual cortex when no covariates were included in the analysis. However, when controlling for the effects of head motion and fMRI protocol, there were not significant results.

While there are many studies reporting significant findings in the field of FC as a predictor of dementia,^11,14,16,21–24,32,34,43–45^ there are also negative studies.^13,17^ A combination of small sample sizes, heterogeneous analytical methods, unknown effect sizes and absence of power analyses may have contributed to our and others’ negative findings.

The post hoc analysis with less strict voxel-level threshold and without control for covariates yielded three clusters in the occipital lobe showing patterns of FC associated with future conversion to AD dementia. This finding did not survive the analyses that controlled for covariates, which may indicate that the findings were confounded by these variables or that the sample and the effect sizes were too small. Interestingly, there is a surprisingly large number of reports with significant FC findings in the occipital cortex in studies involving conversion from MCI to dementia and we are aware of twelve such studies.^11,14,16,21–24,32,34,43–45^ While all included in the preprocessing step some strategy to limit motion-related noise, we found just one study among these twelve that controlled for head motion as a covariate in the group-level statistical analysis.^43^ Another potential confounder is baseline cognition since converters as a group often perform worse at baseline (as we found in the present study); in this regard, out of these twelve FC studies that reported occipital findings, only one study explicitly included a cognitive score (MMSE) as a covariate in its group-level statistical model.^32^ Finally, regarding fMRI protocols, we are aware of three studies that used data from more than one fMRI protocol and reported findings in the occipital lobe and none of these controlled for different fMRI protocols.^16,32,45^

Controlling for different variables appears to influence the associations between FC and future conversion to dementia to varying degrees: the highest decrease in effect size of the association between FC patterns in the occipital clusters and conversion was observed when controlling for baseline cognition, head motion and fMRI protocol. On the other hand, amyloid burden did not seem to have an impact on this effect, despite higher baseline amyloid burden on the MCI-C group. These findings should be taken with considerable caution, given the small sample size and the fact that the three clusters from which these effect size estimates were derived did not remain statistically significant after controlling for these covariates. Effect sizes in neuroimaging studies are often modest and small sample sizes increase the likelihood of inaccurate effect size estimates.^46^

These previous findings in the occipital lobe may thus be at least partially explained by insufficient control of covariates. At the same time, considering how common such findings are, there is also a chance that some real signal is present. In spite of this, the visual cortex is often excluded from analysis.^17,18^ which is not surprising since the occipital lobe is classically known to be spared in the early stages of AD.^47–49^ However, the ubiquitous presence of the occipital lobe among the results, in combination with the broader literature on FC and prior research on networks derived from gray matter,^50^ white matter,^12,51^ glucose metabolism^52^ and amyloid deposition^53,54^ underscores the importance of including the occipital lobe in the analyses even in studies of early-stage AD.

At the analytical core of this study was MDMR, the statistical approach that allowed us to investigate voxelwise patterns of FC associated with future conversion to AD. MDMR has been applied in widely different fields such as genetics,^55^ structural connectivity in AD^56^ and attention-deficit/hyperactivity disorder.^30^ With MDMR there is no need for dimensionality reduction steps, no selection of a potentially arbitrary number of components and no connectivity matrix binarization, so the loss of data is reduced. Moreover, analysis can be performed voxelwise, without parcellating the brain or reducing spatial resolution. Although voxelwise connectivity analysis can be computationally intensive due to the very large numbers of pairwise connections, the current study exemplifies that it is a feasible approach when MDMR is employed. With the release of MDMR library for R the method has become widely accessible.^41^ One limitation that is intrinsically related to this analytical choice is that we have not quantified the stability of p-values at the chosen number of permutations. Although performing 10,000 permutations for each cortical voxel is already computationally demanding, it may be possible that an even higher number would be required to reach tighter p-value stability. Alternatively, if p-value stability could be reached with less permutations, computational resources could be spared. This is a limitation that we share with other studies applying this method^30,57^ and a potential avenue for future research.

Among the strengths of this study, the sample selection process where all MCI were amyloid positive is a highlight. In the field of AD, avoiding the inclusion of participants without AD-related pathology is an advantage since AD itself is already heterogeneous.^58^ By making sure that the whole sample was amyloid positive, we further increased the chance that all converters suffered from dementia due to AD while at the same time making sure that they were compared to peers that were also amyloid positive but did not convert to dementia. Limiting to AD-specific differences in the sample, however, suggests that findings would not generalize to amyloid negative MCI or MCI with mixed biomarker status who are often seen in clinical settings.

There are a number of limitations in our study. Although based on the large ADNI database, the careful sample selection also meant a relatively small sample. This has potentially decreased the statistical power of the study and led to negative results. Additionally, we have not performed power analysis, and it is very possible that with our sample size the study was underpowered to detect robust functional connectivity differences and increases the risk that the findings in the occipital lobe are not generalizable. The size of the spatial smoothing kernel affects the cluster-size threshold because the threshold calculation is based on the degree of spatial smoothing (AFNI's 3dClustSim). The relatively modest smoothing kernel used in the present study (4 mm at FWHM), although chosen to benefit from potentially better spatial specificity, may also influence the power of the study to yield statistically significant results (following previous recommendations, no post hoc change in the kernel size was performed).^40^ This study reinforces the notion that larger sample sizes are fundamental, even if this requires broader selection criteria.

In conclusion, no significant association between FC and future conversion to dementia in MCI was found in the primary analysis while a more relaxed statistical threshold yielded regions in the occipital cortex with FC changes related to disease progression. Occipital FC differences, however, were not robust to methodological and severity factors. Many previous studies have reported findings in the occipital cortex but almost none of them controlled for these covariates. Future studies should focus on larger samples and should include potential confounding variables in the main group-level analyses, especially baseline cognition, head movement during fMRI acquisition and, when appropriate, different fMRI protocols. Finally, this study supports the feasibility of conducting whole-cortex, voxel-based, pairwise FC analysis without spatial downsampling or parcellation schemes when integrated with MDMR.

## Supplemental Material

sj-docx-1-alz-10.1177_13872877261477016 - Supplemental material for Progression from mild cognitive impairment to dementia in Alzheimer's disease: Whole cortex voxelwise functional connectivity analysis with multivariate distance matrix regression Supplemental material, sj-docx-1-alz-10.1177_13872877261477016 for Progression from mild cognitive impairment to dementia in Alzheimer's disease: Whole cortex voxelwise functional connectivity analysis with multivariate distance matrix regression by Luiz Kobuti Ferreira, Eric Westman, Lars-Olof Wahlund, Rosaleena Mohanty and in Journal of Alzheimer's Disease
