# Supplementary material for: Progression from mild cognitive impairment to dementia in Alzheimer's disease: Whole cortex voxelwise functional connectivity analysis with multivariate distance matrix regression
Source: J Alzheimers Dis. 2026 Aug 12;113(3):1384–95. doi: 10.1177/13872877261477016 (PMC13583079; doi:10.1177/13872877261477016)
Supplement: Supplementary file 1 [file sj-docx-1-alz-10.1177_13872877261477016.docx]

**Supplemental Material**

**Progression from mild cognitive impairment to dementia in Alzheimer’s disease: Whole cortex voxelwise functional connectivity analysis with multivariate distance matrix regression**

In order to verify if the results are consistent using another distance metric to calculate the distance matrix, we applied the Euclidean distance instead of the Manhattan distance when checking for the association between future conversion to dementia and baseline FC. Very similarly to the results obtained with Manhattan distance, we found two clusters in the right occipital cortex. These two clusters overlap with two of the three clusters found using Manhattan as the distance metric (Supplemental Table 1 and Supplemental Figure 1).

**Supplemental Table 1.** MDMR, Euclidean distance to calculate the FC distance matrix: clusters with differences in FC pattern between MCI-C and MCI-NC

| Cluster nr | Size  (n voxels) | Brain area | Brodmann area | Peak coordinates  (MNI) | p (FWE) |
| --- | --- | --- | --- | --- | --- |
| 1 | 38 | Postero-lateral visual cortex V2, R | 18 | 35, -97, -7 | 0.0015 |
| 3 | 23 | Postero-lateral visual cortex V2, R | 18 | 20, -101, 16 | 0.0287 |

Significant clusters at voxel-level p < 0.01 and cluster-level p < 0.05, when performing MDMR analysis with Euclidean distance. Peak coordinates in the LPI order, MNI space. FC: functional connectivity; MCI-C: mild cognitive impairment-converters; MCI-NC: non-converters; R: right; p (FWE): p-value corrected for family-wise error at the cluster-level.

**Supplemental Figure 1.** Regions with FC patterns associated with future conversion from MCI to dementia due to AD (distance matrix calculated with Euclidean distance)

Clusters with significantly different patterns of cortical FC at voxel-level p < 0.01 and cluster-level p < 0.05, corrected for multiple comparisons, from the MDMR analysis comparing MCI-C and MCI-NC using Euclidean distance to calculate the distance matrix. The clusters are identified with matching numbers as in Supplemental Table 1 and are overlaid on a 3D-rendered MNI152 template using MRIcroGL (nitrc.org/projects/mricrogl). FC: functional connectivity; MCI-C: mild cognitive impairment-converters to Alzheimer’s disease dementia; MCI-NC: mild cognitive impairment-nonconverters to Alzheimer’s disease dementia; MDMR: multivariate distance matrix regression; L: left; R: right
